# Amino-Functionalized Cellulose Nanofiber/Lignosulfonate New Aerogel Adsorbent for the Removal of Dyes and Heavy Metals from Wastewater

**DOI:** 10.3390/gels9020154

**Published:** 2023-02-14

**Authors:** Islam Elsayed, Gregory T. Schueneman, Emad M. El-Giar, El Barbary Hassan

**Affiliations:** 1 Department of Sustainable Bioproducts, Mississippi State University, Starkville, MI 39762, USA; 2 Department of Chemistry, Faculty of Science, Damietta University, New Damietta 34517, Egypt; 3 USDA Forest Service, Forest Products Laboratory, Madison, WI 53726, USA; 4 School of Sciences, University of Louisiana at Monroe, Monroe, LA 71209, USA

**Keywords:** cellulose nanofibers, lignosulfonates, dyes, heavy metals, wastewater treatment

## Abstract

Due to the increasingly widespread water pollutants and the high cost of treatment methods, there is a demand for new, inexpensive, renewable, and biodegradable adsorbent materials for the purification of wastewater contaminants. In this study, a new biocomposite aerogel (Amf-CNF/LS) was prepared using a chemically cross-linking method between the amino-functionalized cellulose nanofibers (Amf-CNF) and lignosulfonates (LS). The physical and chemical properties of the prepared aerogel were investigated using several techniques including elemental analysis, scanning electron microscopy (SEM-EDS), Fourier transform infrared spectroscopy (FTIR), thermal gravimetric analysis (TGA), and N_2_ adsorption-desorption analysis. The Amf-CNF/LS aerogel was then applied for the removal of methylene blue (MB), rhodamine B dye (RhB), and the heavy metal cadmium ion (Cd^2+^) from synthetic wastewater solutions. The adsorption parameters controlling the adsorption process including the pH, contact time, adsorbent dosage, and adsorbate concen-tration were optimized. High adsorption kinetics and isotherms were observed, with the adsorption isotherms of the Amf-CNF/LS aerogel fitting the Langmuir model with maximum adsorption capacities of 170.94, 147.28, and 129.87 mg/g for MB, RhB, and Cd^2+^, respectively. These results show that Amf-CNF/LS aerogel is a promising green and inexpensive adsorbent for MB, RhB, and Cd^2+^ removal from wastewater.

## 1. Introduction

Various industries that widely use dyes or heavy metal chemicals (e.g., textile, plastic, rubber, paper, cosmetics, electrolysis, electroplating, pesticides, photography, etc.) discard huge quantities of wastewater that still contain some residual dyes and heavy metal contaminants [1,2,3,4]. Methylene blue (MB) and Rhodamine B (RhB) are among the most harmful water pollutants arising mainly from the textile, plastic and dye industries. Both dyes can cause serious problems in the health of the human beings due to their high toxicity and accumulation in the environment [5].Therefore, there is an urgent need to remove and separate these contaminants from water before discharge. Currently, many purification methods have been adopted to remediate wastewater with heavy metals and dye contaminants including chemical reduction, ion exchange, precipitation, flocculation, coagulation, membrane filtration, and adsorption [6,7]. In addition, different dye-containing wastewater treatment methods have been reported, mainly including activated carbon [8], inorganic clay and bentonite [9], zeolite materials [10], synthesized polymers [11], and nanocomposites [12]. Among the previously mentioned wastewater purification methods, adsorption is recognized as one of the most effective and efficient physicochemical methods for wastewater remediation due to its low cost, high efficiency, easy operation, and no additional pollution [13,14,15].

Biorenewable polymers from different resources have gained significant attention from researchers owing to their advantages such as biodegradability, high availability, low cost, eco-friendly, etc. Lignocellulosic biopolymers have exhibited an enormous potential as a replacement for the traditional synthetic polymers to remove contaminants from water [16,17]. Biopolymeric hydrogels (aerogels in a dry form) are considered among the most effective materials for environmental, biomedical, and biological applications owing to their tunable properties [18,19,20]. The chemically cross-linked hydrogels are known as permanent hydrogels due to the strong bonding between the various polymeric networks of the hydrogels [21]. The preparation and characterization of cellulose-lignin hydrogels using epichlorohydrin as a cross linker have been reported [22,23].

Among lignocellulosic biomass components, cellulose is considered one of the most promising natural organic biopolymers due to its abundance, biodegradability, renewability, and low cost. Moreover, it is the most unfailing source of raw material for the increasing demand of environmentally friendly and biocompatible products [24]. Beside cellulose, lignin is a significant constituent of natural lignocellulosic polymers [25,26,27]. Lignin, as a natural aromatic biopolymer, is considered the second most abundant natural polymer. In addition, lignin is also one of the popular environmentally friendly biorenewable resources [26]. Moreover, the importance of lignin is a reflection of many advantages such as being an antioxidant, an antimicrobial agent, being in high abundance, biodegradable, carbon dioxide neutral, etc. [25].

Lignin is produced in large quantities, reaching millions of tons, annually in the form of lignosulfonate which makes it an important renewable aromatic source [28]. The abundance of phenolic hydroxyl and carboxyl groups in the lignin network makes lignin one of the best platform sources of active sites for the adsorption of dyes and heavy metals [29,30]. Annually, pulping industries produce more than 50 million tons of lignin worldwide. About 90% of this quantity is burned for power generation and the remainder is used for the production of various products [31]. The high availability of lignin attracted scientists’ interest in exploring more valuable applications for it such as developing lignosulfonates as a natural adsorbent for the removal of wastewater contaminants.

In the past, lignosulfonates have been utilized as an important material for the production of many products such as stabilizing agents [32], lubricants [33], coatings [34], surfactants [35], superabsorbent hydrogels [36], and others [37,38]. Therefore, focusing on using lignosulfonate biopolymers in the preparation of new functional materials for wastewater treatment will solve the waste disposal problem as well as increase its economic value. Yu et al. [39] reported the synthesis of highly efficient bioabsorbent hydrogels by grafting acrylic acid on the surface of lignosulfonate for the removal of the industrial cationic methylene blue (MB) dye with adsorption capacity ~2013 mg/g. Furthermore, Zhao et al. [40] explored the use of lignosulfonate-graft-poly-(acrylic acid)/hydroxyethyl cellulose semi-interpenetrating hydrogels for the extraction of organic dye contaminants from aqueous solutions. Panzarasa et al. [41] described a simple way to synthesize a hybrid adsorbent material by combining lignin and lignosulfonates with poly(ethylene-alt-maleic anhydride) for water purification. The adsorption efficiency for MB was rapid and achieved more than 99% at a wide range of pH (2–12), coupled with noticeable stability and insolubility [41]. Lignin-based Fe_3_O_4_@lignosulfonate/phenolic microspheres were successfully synthesized by Wang et al. [42] for the removal of cationic dyes from aqueous solutions. The Langmuir model has been successfully applied to the adsorption equilibrium data resulting in a maximum monolayer adsorption capacity of 283.6 mg/g [42]. Recently, Liu et al. [30] prepared a hydrogel by cross linking polymerization with acrylamide (AM) and sodium lignosulfonate for the removal of cadmium and tetracycline(TC) from water samples.

Considering the various advantages of nanocellulose, lignosulfonate, and chemically cross-linked hydrogels, the current study focused on preparing a new aerogel adsorbent by chemical cross-linking between lignosulfonates and the amino-functionalized cellulose nanofiber (Amf-CNF) for the removal of MB, rhodamine b dye (RhB), and cadmium ions (Cd^2+^) from wastewater. The prepared aerogel was characterized using several characterization techniques including elemental analysis, scanning electron microscopy (SEM-EDS), Fourier transform infrared spectroscopy (FTIR), thermal gravimetric analysis (TGA), and N_2_ adsorption-desorption analyses. The adsorption parameters (pH, adsorbent dosage, dye concentration, contact time, and temperature) were optimized. In addition, the adsorption kinetics and isotherms were evaluated.

## 2. Results and Discussion

### 2.1. Point of Zero Charge (PZC)

**Figure 1:**
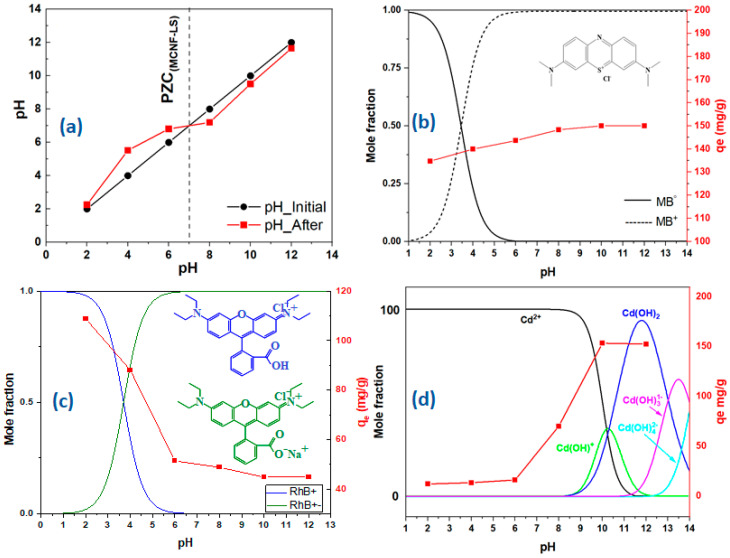
(**a**) Point of zero charge (PZC) determination curve of Amf-CNF/LS, (**b**–**d**) speciation curves at different pH values for MB, RhB, and Cd^2+^ adsorptions, respectively.

The adsorption phenomena of adsorbent materials are significantly influenced by the surface charge. As shown in Figure 1a, the point of zero charge (PZC) study is essential to provide the surface charge information of the adsorbent at different pH levels. The PZC for Amf-CNF/LS was detected at pH = 7.03. In solutions with pH values of less than 7.03, the Amf-CNF/LS surface was positively charged. On the other hand, with pH values higher than 7.03, the Amf-CNF/LS surface charge becomes negative, which could be attributed to the existence of the sulfonate groups (−SO_3_ˉ) on the lignosulfonate surface. Therefore, performing the adsorption experiments in a solution with pH values higher than 7.03 will create a negative charge on the adsorbent surface which strengthens the electrostatic interactions with cationic organic and inorganic contaminants [43,44].

The effects of pH on the adsorption capacities are shown in Figure 1b–d with the speciation curves of MB, RhB and Cd^2+^ contaminates. As depicted in Figure 1b, the concentration of the MB cationic form gradually increased from pH 1 to a maximum at pH 6 and remained the most abundant species up to pH 14. Matching this behavior, the adsorption capacity of MB rose from 134.72 mg/g at pH 2 to 150 mg/g at pH 10 and remained constant at higher pH. Taking the industrial preference to work closer to a neutral pH, the adsorption study of MB was performed at pH 8. For the RhB contaminant (Figure 1c), the speciation curve reveals a decrease of the concentration of the RhB cationic form from 100% at pH 1 to less than 1% at pH 6 with the increasing predominance of the RhB form that contains anionic and cationic sites from 0% at pH 1 to 100% at pH 6. Thus, RhB adsorption capacity has a sharp decline from 108.85 mg/g at pH 2 to 51.36 mg/g at pH 6 then a slight decrease to 44.8 mg/g at pH 10. Accordingly, pH 6 was selected for the RhB adsorption studies. As depicted in Figure 1d, the concentration of Cd^2+^ was the most abundant species (100%) until pH 8 then gradually decreased to 0% at pH 11.5 with increasing concentrations of other cadmium forms. At pH 7.5, turbidity was observed in the solution which is attributed to the formation of cadmium hydroxide (Cd(OH)_2_). The adsorption capacity of Cd^2+^ increased slightly from 2.76 mg/g at pH 2 to 6.83 mg/g at pH 6 followed by a jump to 143.89 mg/g at pH 10 and higher due to the precipitation of cadmium in the form of Cd(OH)_2_. Considering these observations, the adsorption studies of cadmium contaminant were conducted at pH 7.

### 2.2. Characterization of Amf-CNF/LS Aerogel

The amination reaction for CNF was monitored via the nitrogen content in the formed aerogel using combustion elemental analysis for CNF, Amf-CNF, and Amf-CNF/LS as shown in Table 1. The initial nitrogen content in CNF was 0.06% and after the amination reaction the nitrogen content increased to 2.09%, which confirmed the successful amination of CNF to produce Amf-CNF and the presence of tertiary amines groups on CNF surface. The nitrogen content slightly declined in the Amf-CNF/LS aerogel due to the presence of lignin which contains high contents of carbon, hydrogen, oxygen, and sulfur.

**Figure 2:**
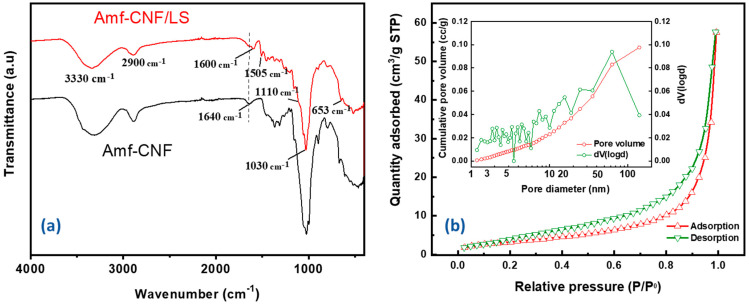
(**a**) FTIR spectra of Amf-CNF and Amf-CNF/LS aerogels. (**b**) Nitrogen adsorption-desorption isotherms at −196 °C and BJH average pore size distribution of the Amf-CNF/LS aerogel.

FTIR spectra were recorded for both Amf-CNF and Amf-CNF/LS to explore the chemical function groups (Figure 2a). The Amf-CNF/LS FTIR spectrum shows a band at 1600 cm^−1^ which is attributed to the stretching vibration peak of the C=C group in the lignin aromatic ring, while the peak at 1505 cm^−1^ is the characteristic absorption peak of the aromatic skeletal vibrations. The absorption peak of the bending vibration of the phenolic hydroxyl groups appeared at 1110 cm^−1^ and the peaks at 653 cm^−1^ and 1030 cm^−1^ are the symmetrical stretching vibration of S-O and S=O bonds of the sulfonate groups, respectively. The Amf-CNF sample exhibited an absorption peak at 1640 cm^−1^ that corresponds to the vibration of the N-C bond of the quaternary ammonium groups [45,46,47]. These FTIR results clearly indicate the successful addition of tertiary amine on the surface of CNF and the ionic interaction between lignosulfonate and Amf-CNF forming an Amf-CNF/LS aerogel.

The Brunauer−Emmett−Teller (BET) and Barrett−Joyner−Halenda (BJH) methods were applied to measure the surface area and pore size distribution for the nitrogen adsorption-desorption isotherm, respectively. As shown in Figure 2b, the Amf-CNF/LS aerogel shows a type IV_a_ N_2_ adsorption-desorption isotherm with a hysteresis loop indicating the presence of both mesopores and macropores. The BET surface area of the Amf-CNF/LS aerogel was 12.13 m^2^/g, the total pore diameter was 29.4 nm, and the total pore volume 0.089 cc/g for pores smaller than 267.7 nm diameter. The presence of a hierarchical mesoporous−macroporous structure in the adsorbent is vital because, during water purification, the macropores allow faster molecular diffusion and mass transfer (mainly for big molecules) and the mesopores produce more adsorption active sites for contaminant removal [48].

**Figure 3:**
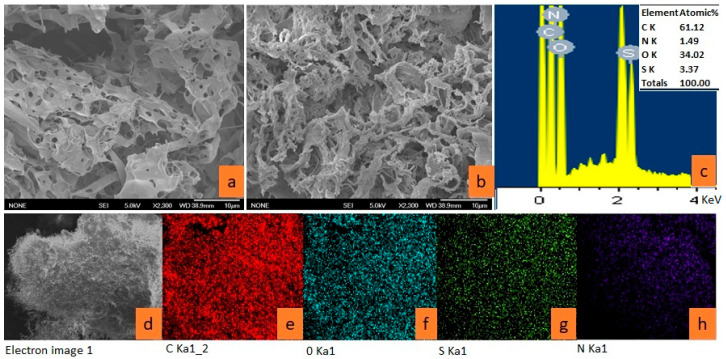
(**a**,**b**) SEM images of the amino-modified cellulose nanofibers (Amf-CNF) and (Amf-CNF/LS) aerogels, (**c**) The metal content curve of Amf-CNF/LS aerogel. (**d**–**h**) SEM-EDS images.

Figure 3 shows the morphological structures for the Amf-CNF and Amf-CNF/LS aerogels examined by the EDX-SEM. The SEM images in Figure 3a,b show that both Amf-CNF and Amf-CNF/LS aerogels have mesoporous structures with a film-like or net-like cell morphology which is important for the adsorbent in water purification [48]. In addition, the elemental content curve in Figure 3c and EDS images (Figure 3d–h) prove the presence and good dispersion of tertiary amine and sulfonate groups on the Amf-CNF/LS aerogel surface.

### 2.3. Adsorption Study

#### 2.3.1. Adsorption Kinetics

**Figure 4:**
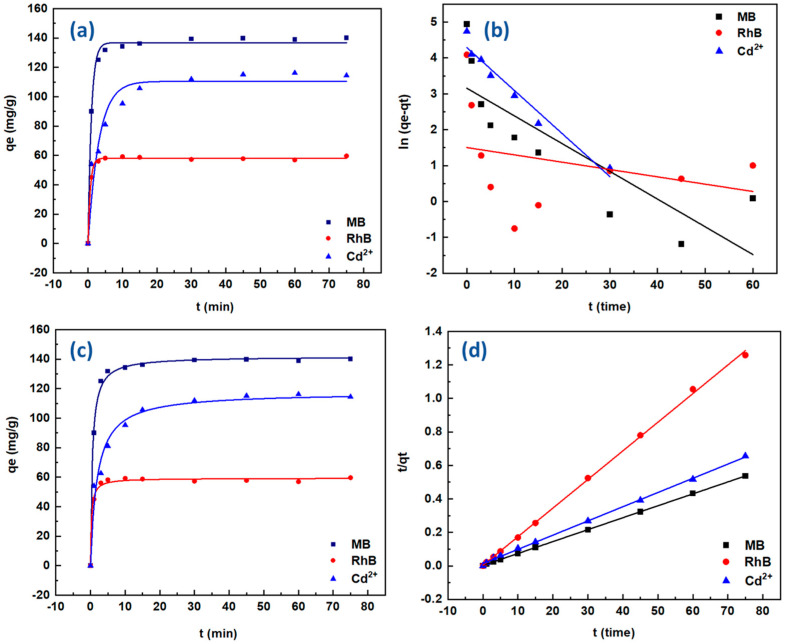
(**a**,**b**) The adsorption results with non-linear and linear fittings of the pseudo-first order kinetic model, respectively. (**c**,**d**) The adsorption results with non-linear and linear fittings of the pseudo-second order kinetic model, respectively.

The effects of the contact adsorption time on the adsorption capacities of RhB, MB, and Cd^2+^ at different initial concentrations, ranging from 100 ppm for RhB to 150 ppm for both RhB and Cd^2+^, are displayed in Figure 4. It is obvious that (Figure 4a,c) the adsorption capacities of both MB and RhB increased rapidly in the first 2 min and reached the maximum adsorption capacity (140.01 mg/g for MB and 59.61 mg/g for RhB) within 5 min for MB and 3 min for RhB. On the other hand, Cd^2+^ adsorption also increased rapidly but at a somewhat slower pace than MB and RhB in the first 10 min and reached the highest adsorption capacity (116.08 mg/g) within 15 min. The high adsorption rates for the three contaminants are attributed to the prevalent existence of strong interactions between the anionic sulfonic groups on the surface of the Amf-CNF/LS adsorbent and the cationic adsorbates. The adsorption kinetic data for MB, RhB, and Cd^2+^ were investigated using two kinetic models, namely pseudo-first order and pseudo-second order, and both models’ adsorption kinetic parameters are listed in Table 2. The adsorption kinetic data were fitted better to the pseudo-second order model than to the pseudo-first order model due to the strong matching between the calculated (q_e_·_cal_) and the experimental (q_e_·_exp)_ adsorption capacities, higher R-Square (COD, R^2^ > 0.99), and lower reduced Chi-Sqr (χ^2^) values. In addition, there is a perfect fit of the experimental adsorption points on the linear form of the pseudo-second order model. The pseudo-second order’s adsorption kinetics propose a bimolecular adsorption mechanism, where both surface functional groups of the adsorbent and adsorbate contribute to the chemisorption process’s rate determining step [49,50,51].

#### 2.3.2. Adsorption Isotherm

**Figure 5:**
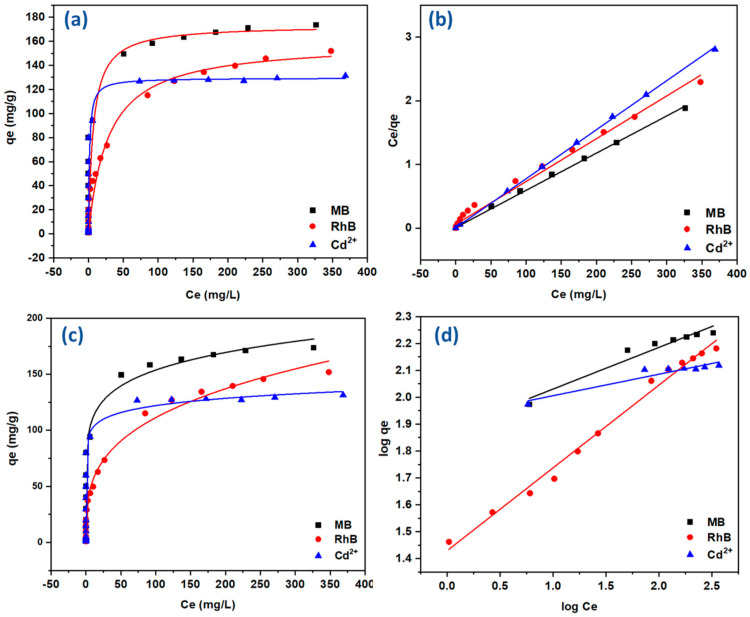
(**a**,**b**) The experimental adsorption results with non-linear and linear fittings of the Langmuir isotherm model. (**c**,**d**) The experimental adsorption results with non-linear and linear fittings of the Freundlich isotherm model.

The isotherms for the adsorption of MB, RhB, and Cd^2+^ onto Amf-CNF/LS adsorbent are shown in Figure 5. The Langmuir and Freundlich isotherm models have been applied to simulate the equilibrium adsorption isotherm data. Based on the results listed in Table 3, the data were fitted with the Langmuir model much better than with the Freundlich model owing to the high R-Square (COD, R^2^ > 0.99) and smaller reduced Chi-Sqr (χ^2^) values which indicate higher accuracy with the Langmuir model. The maximum Langmuir adsorption capacities for MB, RhB, and Cd^2+^ were estimated to be 171.23, 148.81, and 129.87 mg/g, respectively. The Langmuir isotherm model assumes a homogenous monolayer chemisorption coverage with no interactions between adsorbate molecules, indicating chemical interactions between the Amf-CNF/LS aerogel and the adsorbates. The low (R^2^) value for the Freundlich adsorption model of Cd^2+^ (0.686) compared with that of dyes (R^2^ > 0.94) confirms that the adsorption of Cd^2+^ is a homogenous monolayer adsorption. The adsorption capacities of different lignin-based adsorbents towards MB, RhB, and Cd^2+^ contaminates are presented in Table 4. The adsorption capacity of Amf-CNF/LS was relatively higher than the adsorption capacities of most lignin-based adsorbents applied at similar pH values used in this study. Some expensive, non-biodegradable adsorbents applied at extreme low or high pH values [42,52,53,54,55] show relatively higher adsorption capacity values than Amf-CNF/LS.

#### 2.3.3. Adsorption Mechanism

**Figure 6:**
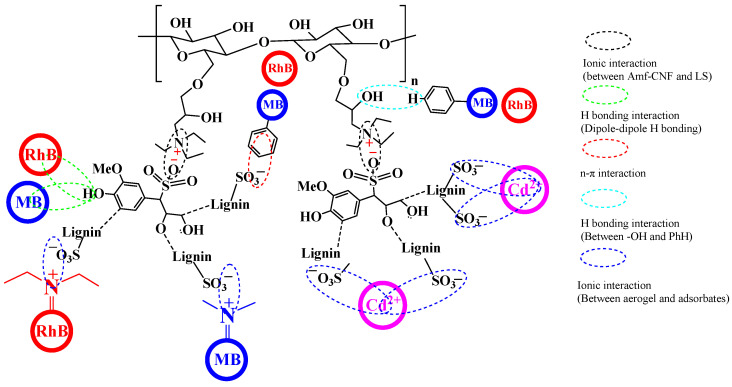
Illustration of the possible adsorption mechanism between the Amf-CNF/LS aerogel and MB, RhB, and Cd^2+^ adsorbates.

The adsorption mechanism of MB, RhB, and Cd^2+^ onto the surface of Amf-CNF/LS adsorbent is shown in Figure 6. It is clear from the figure that the adsorption mechanism is mainly governed by both the ionic and H bonding interactions between the aerogel and the adsorbates [68,69]. The negative charge on the aerogel sulfonate groups (−SO_3_^−^) can easily interact with the positive charge of the cationic amino groups (−NH_3_^+^) of the dyes and the positive charge on the Cd^2+^. This electrostatic interaction represents the main driving force for the adsorption of both dyes as well as the Cd^2+^ on the surface of the aerogel. The aerogel sulfonate groups (−SO_3_^−^) can also form n-π interactions with the aromatic rings of MB and RhB dyes. Furthermore, the possible hydrogen bonding between the free hydrogen and oxygen of both aerogel adsorbent and dyes can also increase the adsorption process.

#### 2.3.4. Recyclability

After the adsorption process, the Amf-CNF/LS aerogel will undergo chemical treatment to remove contaminants and reuse it for further water treatment operations. The removal process of the contaminants will be performed as follows: the aerogel loaded cationic dyes will be treated with ethanol at pH 2 using 1 M HCl, while the aerogel loaded cadmium will be mixed with 1 M HCl and kept in ultrasound or in an incubator shaker for 60 min. The solid aerogel will be separated, washed with distilled water, and applied in repeated adsorption cycles.

## 3. Conclusions

A new biocomposite aerogel (Amf-CNF/LS) was successfully prepared through the ionic interaction between tertiary amine groups of the amino-functionalized CNF and sulfonate groups of the lignosulfonate. The aerogel was characterized using different analytical techniques (FTIR, SEM-EDS, elemental analysis, and N^2^ adsorption-desorption isotherms) and employed for the adsorption of MB, RhB, and Cd^2+^ from synthetic wastewater solutions. The obtained results indicated that pH, contact time, adsorbent dosage, and adsorbate concentration were key parameters for the adsorption process. The adsorption process proceeded rapidly within 5 min and the kinetic data followed the pseudo-second order model, proposing a bimolecular adsorption mechanism governed by the ionic interaction between the sulfonate groups on the aerogel adsorbent surface (Amf-CNF/LS) and the cationic groups on the adsorbate surface. Adsorption isotherms results fitted with the Langmuir model much better than the Freundlich model, indicating homogenous monolayer chemical interactions between aerogel and adsorbates functional groups with no interactions between adsorbate molecules. The maximum adsorption capacities for MB, RhB, and Cd^2+^ were 171.23, 148.81, and 129.87 mg/g, respectively. The maximum Langmuir adsorption capacities were greater than most of the previously reported biobased materials.

## 4. Materials and Methods

### 4.1. Materials

**Figure 7:**
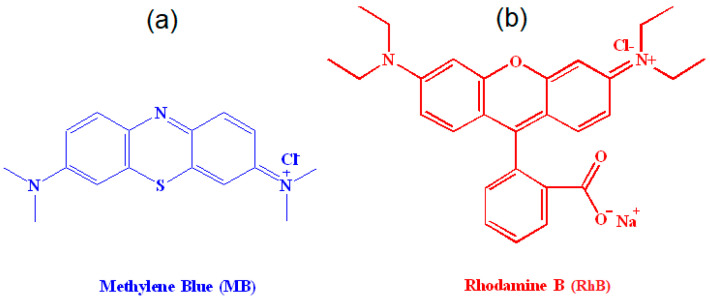
The chemical structures of (**a**) methylene blue and (**b**) rhodamine B.

A 3% aqueous slurry of Cellulose nanofiber (CNF) was obtained from the UMaine Process Development Center. Sodium lignosulfonate (LS) TECH grade was purchased from Pfaltz & Bauer Inc. Epichlorohydrin (EPH), 99% acroseal was purchased from Acros Organics. Triethylamine (TEA), 99% reagent grade and hydrochloric acid (HCl), 37.1% certified ACS plus were purchased from Fisher Chemical. Sodium hydroxide bioxtra, ≥98% pellets (anhydrous), methylene blue (MB, Sigma-Aldrich, CAS: 61-73-4, empirical formula C_16_H_18_ClN_3_S, MW: 319.85 g/mol, Figure 7a), and Rhodamine B (RhB, Sigma-Aldrich, CAS: 81-88-9, empirical formula C_28_H_31_ClN_2_O_3_, MW: 479.01, Figure 7b) were obtained form Sigma-Aldrich (St. Louis, MO, USA). The Thermo Scientific E-pure A water purification system was used to provide high purity deionized water (17.8 MΩ·cm).

### 4.2. Preparation of Regenerated Cellulose

In a 250-mL volumetric flask, about 10 g of NaOH pellets were added to a 100 mL of 3% aqueous slurry of CNF with vigorous mechanical stirring until complete dissolution. The mixture was then placed in a cooled bath at −5 °C until a transparent cellulose gel was formed. The gel was diluted with deionized water and neutralized using a dilute HCl solution, followed by 20 min of mechanical blending at 25,000 rpm using a high-speed blender (Vitamix 5200, Cleveland, OH, USA) to obtain a good dispersed regenerated cellulose. Finally, the cellulose was washed and collected through vacuum filtration using a Whatman (grade 42) filter paper. The pH of the cellulose was 7.0.

### 4.3. Preparation of Amino-Modified Cellulose Nanofibers (Amf-CNF)

In a 250-mL volumetric flask, one third of the previously prepared cellulose was dispersed in 100 mL of deionized water. Then, a mixture of 7.8 mL of EPH and 14 mL TEA were added drop by drop with continuous stirring. The mixture was then refluxed at 80 °C for 24 h with continuous magnetic stirring. The mixture was neutralized, vacuum filtered, and washed several times with deionized water until water pH was attained. A portion of the product was freeze dried to produce an aerogel for use in the characterization experiments.

### 4.4. Preparation of Amino-Modified Cellulose Nanofibers/Lignosulfonate Aerogel (Amf-CNF/LS)

**Scheme 1:**
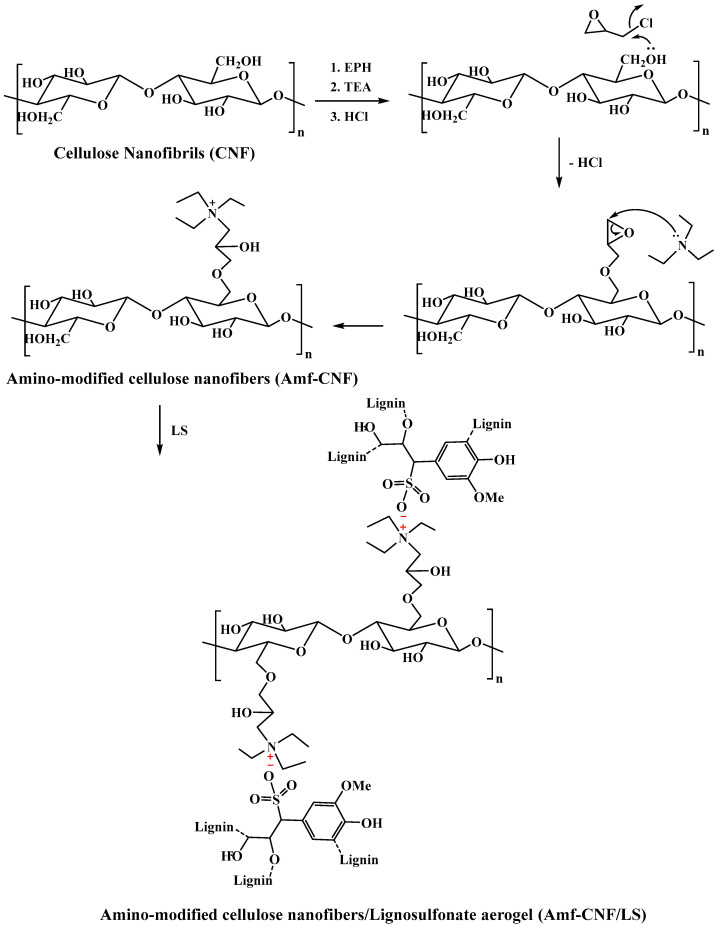
The proposed mechanism for the Amf-CNF/LS aerogel formation.

In a 500 mL beaker, the prepared amino-modified cellulose nanofibers were dispersed in 100 mL of deionized water. A 100 mL of 3% lignosulfonate solution was then added drop by drop with magnetic stirring for 30 min and the pH of the mixture was adjusted to 9.0 using NaOH solution. The product was collected by vacuum filtration, washed several times with deionized water until water pH was attained. Finally, the sample was dried in a freeze drier until an aerogel was obtained. The proposed mechanism for the Amf-CNF/LS aerogel formation are shown in Scheme 1.

### 4.5. Characterization of Amf-CNF/LS Aerogel

The FTIR spectra were collected using a Thermo Scientific Nicolet iS50 FTIR spectrometer in the 4000 to 400 cm^−1^ range. The elemental composition (carbon, hydrogen, nitrogen, and oxygen) was determined using a CE-440 Elemental Analyzer (Exeter Analytical Inc., North Chelmsford, MA, USA). The aerogels surface morphology was determined by a FE-SEM (JEOL JSM-6500F, Tokyo, Japan). Aerogel samples were sputter-coated with 15 nm platinum and imaged at a 5 keV accelerating voltage. A Quantachrome Autosorb iQ gas sorption analyzer (Quantachrome ASIQC0500-5, Boynton Beach, FL, USA) was used to measure the specific surface area, total pore volume, and average pore diameter using nitrogen adsorption-desorption isotherms at −196 °C. Each aerogel sample was degassed for 3 h at 105 °C under a vacuum. The Brunauer−Emmett−Teller (BET) and Barrett−Joyner−Halenda (BJH) methods were applied to measure the specific surface area and pore size distribution, respectively.

#### 4.5.1. PZC Measurements

The pH drift method was applied to measure the PZC of Amf-CNF/LS by keeping the ionic strength constant with 20 mL of 0.01 M NaCl. Briefly, the solution pH was set to a range from 2 to 12, in intervals of 2, using either 0.1 M NaOH or HCl solutions (initial pH). The NaCl solutions were purged with N_2_ before adding the adsorbent to remove dissolved CO_2_. The solutions (20 mL) were equilibrated with 20 mg of adsorbent and agitated in a mechanical shaker (250 rpm) at room temperature for 24 h. Next, the pH of the solutions was measured again (final pH). The PZC was detected by graphically plotting the initial and final pH values.

#### 4.5.2. Effect of pH

The effect of adsorbate solution pH on the adsorption capacity was evaluated by adjusting the pH range from 2 to 10 in intervals of 2. At room temperature, 10 mg of Amf-CNF/LS were added to 10 mL of 150 ppm adsorbate solutions, then the mixture was agitated at 250 rpm in a mechanical shaker for 30 min. The samples were then filtered with a 0.22 µm syringe filter, and the concentration of each adsorbate filtrate was determined. The concentrations of MB and RhB before and after the batch adsorption were determined using an Azzota SM1800PC UV-Vis spectrophotometer at a wavelength of 664 and 554 nm, respectively. The adsorption intensities of MB and RhB were converted to a concentration value by a linear regression curve obtained from the calibration curve over a concentration range of 2−20 mg/L for MB and a concentration range of 2−10 mg/L for RhB. The concentration of Cd^2+^ after contaminants uptake was measured using a Shimadzu AA-7000 atomic absorption spectrophotometer by a linear regression curve obtained from the calibration curve over a concentration range of 20−500 mg/L. All adsorption experiments were performed with three replicates and the initial pH values of the adsorbate solutions were adjusted using either HCl or NaOH.

### 4.6. Adsorption Experiments

#### 4.6.1. Adsorption Kinitics

The adsorption kinetics of the three contaminants were performed by adding 10 mg of the Amf-CNF/LS sample to 10 mL of either 150 ppm (for MB and Cd^2+^) or 100 ppm (for RhB) of the adsorbate solution. All adsorption experiments were conducted at room temperature and the pH of MB, Cd^2+^, and RhB solutions were 8, 7, and 6, respectively. The samples were agitated at 250 rpm in a mechanical shaker for predefined time intervals ranging from 1 to 75 min. The adsorption capacity (*q*) was determined using the following equation:

(1) $$q=\frac{\left(C_{0}-C_{t}\right)\times V}{m}$$

where *C*_0_ (mg/L) and *C_t_* (mg/L) are the adsorbate concentrations (mg/g) before and after adsorption, respectively, *V* (L) is the adsorbate solution volume, and *m* (g) is the adsorbent’s dry mass. Pseudo-first order and pseudo-second order adsorption kinetic models were applied using the following equations:

(2) $$\ln\left(q_{e}-q_{t}\right)=\ln q_{e}-k_{1}t\;$$

(3) $$\frac{t}{q_{t}}=\frac{1}{k_{2}q_{e}^{2}}+\frac{t}{q_{e}}\;$$

where *q_e_* (mg/g) is the adsorption capacity at equilibrium, *q_t_* (mg/g) is the adsorption capacity at time *t*, *k*_1_ (min^−1^) is the pseudo-first order rate constant, and *k*_2_ (g/mg·min) is the pseudo-second order rate constant.

#### 4.6.2. Adsorption Isotherms

The adsorption isotherms were evaluated following the same method used for the kinetic studies. All adsorption isotherm studies were performed at room temperature at pH 8, 7, and 6 for MB, Cd^2+^, and RhB solutions, respectively. All adsorption isotherms were conducted using different concentration ranges from 1 mg/L to 500 mg/L for 1 h. The Langmuir (Equation (4)) and Freundlich (Equation (5)) adsorption isotherm models were applied using the following equations:

(4) $$\frac{C_{e}}{q_{e}}=\frac{Ce}{q_{m}}+\frac{1}{q_{m}b}$$

(5) $$\log q_{e}=\log k_{f}+\frac{1}{n}\log C_{e}\;$$

where *C_e_* (mg/L) is the adsorbate solution concentration at equilibrium, *q*_e_ (mg/g) is the equilibrium adsorption capacity, *q_m_* (mg/g) is the maximum adsorption capacity, *b* is the Langmuir adsorption constant related to adsorption energy, *K_f_* and *n* are the Freundlich adsorption constants which indicate the capacity and intensity of the adsorption, respectively.

## Figures and Tables

**Table 1:** Elemental analysis and surface area of CNF, MCNF, and AMF-CNF/LS.

Sample	Elemental Analysis				Surface Area (m^2^/g)
	%C	%H	%N	%O	
CNF	42.87	6.27	0.06	50.82	2.82
Amf-CNF	45.15	7.17	2.09	45.59	9.18
Amf-CNF/LS	48.23	6.66	1.58	43.55	12.13

**Table 2:** The adsorption kinetic parameters for MB, RhB, and Cd^2+^.

**Cont.** **Type**	**Pseudo-First-Order**					
	***q*** ***_e_*** **(exp) (mg/g)**	***q*** ***_e_*** **(calc) (mg/g)**	***K*** **_1_** **(min^−1^)**	**R^2^**	**Reduced** **Chi-Sqr (χ^2^)**	**Root-MSE (SD)**
MB	140.01	23.48	0.078	0.694	1.397	1.182
RhB	59.61	4.49	0.020	0.090	2.23	1.492
Cd^2+^	116.08	72.85	0.120	0.959	0.083	0.289
	**Pseudo-Second-Order**					
	***q*** ***_e_* (exp) (mg/g)**	***q*** ***_e_*** **(calc) (mg/g)**	***K*** **_2_** **(min^−1^)**	**R^2^**	**Reduced** **Chi-Sqr (χ^2^)**	**Root-MSE** **(SD)**
MB	140.01	140.45	0.02	0.999	2.09 × 10^−6^	1.44 × 10^−3^
RhB	59.61	58.51	0.061	0.999	1.97 × 10^−4^	1.40 × 10^−2^
Cd^2+^	116.08	117.93	0.004	0.998	5.50 × 10^−5^	7.42 × 10^−3^

**Table 3:** The adsorption isotherm parameters for MB, RhB, and Cd^2+^.

**Cont.** **Type**	**Langmuir Model**				
	**Qmax** **(mg/g)**	***K*** ***_L_*** **(L/mg)**	**R^2^**	**Reduced** **Chi-Sqr (χ^2^)**	**Root-MSE** **(SD)**
MB	171.23	0.76	0.999	3.43 × 10^−4^	0.019
RhB	148.81	0.12	0.989	5.37 × 10^−3^	0.073
Cd^2+^	129.87	1.92	0.999	1.84 × 10^−4^	1.36 × 10^−2^
	**Freundlich Model**				
	***K*** ***_f_***	***n***	**R^2^**	**Reduced** **Chi-Sqr (χ^2^)**	**Root-MSE** **(SD)**
MB	74.98	6.44	0.945	5.76 × 10^−4^	0.024
RhB	26.88	3.25	0.990	7.29 × 10^−4^	0.027
Cd^2+^	84.31	12.51	0.686	2.83 × 10^−4^	0.017

**Table 4:** Adsorption capacity of various lignin-based adsorbents towards MB, RhB, and Cd^2+^ removal.

Adsorbent	Adsorbate	pH	Qmax (mg/g)	Reference
Amf-CNF/LS	MB	8	171.23	This study
Amino-functionalizedTiO_2_/sodium lignosulfonate		11	37	[56]
Sodium alginate/lignin −20% beads		12	254.3	[52]
Activated lignin-chitosan (Lig/CS)		7	36.25	[13]
Lignosulfonate/amino-functionalized SBA-15		9	62.89	[57]
Magnetic chitosan lignosulfonate (MGLS)		10	81.4	[53]
Magnetic chitosan lignosulfonate/graphene oxide		10	253.53	[53]
Fe_3_O_4_@lignosulfonate/phenolic core-shell microspheres		10	283.6	[42]
Amf-CNF/LS	RhB	6	148.81	This study
Magnetic lignosulfonate		2	22.47	[58]
Magnetic lignin microspheres from larch lignin		—	17.62	[59]
Ligno-cellulosic solid wastes		5.7	7.309	[60]
Chitosan–lignosulfonate Composite		7	126.58	[61]
Lignosulfonate/poly acrylic acid/Al hydrogels		7	334.64	[54]
Cross-linked lignin		—	156.4	[62]
Amf-CNF/LS	Cd^2+^	7	129.87	This study
1,2,4-triazole modified lignin-based adsorbent		6	87.4	[63]
Modified lignin from beech and poplar woods		4.5	6.7–7.5	[64]
Kraft lignin		4.5	137.14	[55]
Modified lignin on corncob-based biochar		7	85.65	[65]
Aminated lignin		5	43.2	[66]
lignin hydrogels loaded with nano-FeS		7	215	[30]
Hematite/lignosulfonate composite (HLS)		5	53.65	[67]

## Data Availability

Data are contained within the article.
